# Reluctance of general practice staff to register patients without documentation: a qualitative study in North East London

**DOI:** 10.3399/BJGP.2022.0336

**Published:** 2022-12-13

**Authors:** Kitty Worthing, Pooja Seta, Isa Ouwehand, Anita Berlin, Megan Clinch

**Affiliations:** Centre for Primary Care, Wolfson Institute of Population Health Sciences, Queen Mary University of London, London; Sheffield Teaching Hospitals NHS Foundation Trust, Royal Hallamshire Hospital, Sheffield.; Faculty of Medicine and Dentistry, Queen Mary University of London, London.; Faculty of Medicine and Dentistry, Queen Mary University of London, London.; Community Based Medicine Education, Institute of Health Science Education, Queen Mary University of London, London.; Centre for Public Health & Policy, Wolfson Institute of Population Health Sciences, Queen Mary University of London, London.

**Keywords:** access to health care, general practice, qualitative research, staff attitude, transients and migrants, undocumented immigrants

## Abstract

**Background:**

Lack of access to documentation is a key barrier to GP registration, despite NHS England guidance stating that documents are not required. Staff attitudes and practice regarding registration of those without documentation are under- researched.

**Aim:**

To understand the processes through which registration might be refused for those without documents, and the factors operating to influence this.

**Design and setting:**

Qualitative study conducted in general practice across three clinical commissioning groups in North East London.

**Method:**

In total, 33 participants (GP staff involved in registering new patients) were recruited through email invitation. Semi-structured interviews and focus groups were conducted. Data were analysed using Braun and Clarke’s reflexive thematic analysis. Two social theories informed this analysis: Lipsky’s street-level bureaucracy and Bourdieu’s theory of practice.

**Results:**

Despite good knowledge of guidance, most participants expressed reluctance to register those without documentation, often introducing additional hurdles or requirements in their everyday practice. Two explanatory themes were generated: that those without documents were perceived as burdensome, and/or that moral judgements were made about their deservedness to finite resources. Participants described a context of high workload and insufficient funding. Some felt that GP services should be restricted by immigration status, as is widespread in secondary care.

**Conclusion:**

Improving inclusive registration practice requires addressing staff concerns, supporting navigation of high workloads, tackling financial disincentives to registering transient groups, and challenging narratives that undocumented migrants represent a ‘threat’ to NHS resources. Furthermore, it is imperative to acknowledge and address upstream drivers, in this instance the Hostile Environment.

## INTRODUCTION

Current NHS England guidance states that everyone in England can register with a GP, regardless of immigration status, and that they do not require documentation or an NHS number to do so.^1^ However, it remains routine practice to ask for proof of address and identification. Although the guidance states that asking is permissible, registration cannot be refused because of lack of documents.^1^

Research has consistently shown that people attempting to register with a GP who do not have the requested documentation are often refused registration. Doctors of the World (DOTW), a non-governmental organisation (NGO) that supports service users to register with a GP, has consistently found that <19% of attempts by trained advocates to gain registration for patients without documents by phone are refused.^2^^–^4 Recent ‘mystery shopper’ exercises found that registration is refused in 48%–74% of cases.^5^^–^7 In addition, a review of general practice websites in London found that 75% asked for documentation for registration, with the majority of these framing this as a demand.^8^ Furthermore, qualitative studies have found that those without access to documentation identify this as a barrier to registering with a GP.^9^^,^^10^

Three groups are commonly cited in the literature as facing challenges to access general practice because of a lack of documentation: people who are ‘undocumented’ migrants^2^^,^^9^ (Box 1), those experiencing homelessness,^11^^,^^12^ and Gypsy, Roma, and Traveller (GRT) communities.^5^^,^^6^ While theoretically no NHS services are restricted because of lack of documentation, restrictions based on immigration status do exist in secondary care and affect people who are undocumented as a result of the Overseas Visitor and Migrant NHS Cost Recovery Programme (Box 2).^13^^,^^14^

**Box 1.** People who are undocumented

People who are undocumented refers to those whose immigration status is not recognised, regulated, or documented by the state. Immigration status is often complex and shifting. There are multiple ways in which a person can become ‘undocumented’, including: being born in the UK to undocumented parents; having entered the UK with a visa but having a change in circumstances, or staying beyond the timeframe of the visa; those who entered the UK without a visa, including survivors of modern-day slavery and trafficking; or remaining after an unsuccessful asylum claim. It is difficult to ascertain the number of people who are undocumented, but UK estimates since 2001 range from 120 000 to 1.3 million.^14^ For the purpose of this article the above groups are referred to as people who are undocumented. These are included in a wider group of people who cannot access documentation when requested by GP practices.^13^

**Box 2.** Summary of current NHS restrictions to access to care based on immigration status

Primary care and care provided by accident and emergency departments are free at the point of delivery for all people living in the UK regardless of immigration status. Under the Overseas Visitor and Migrant NHS Cost Recovery Programme, people who are not deemed ‘ordinarily resident’, or have not paid the NHS migrant surcharge, are charged for secondary care at 150% of the actual cost. This includes people who are undocumented, as described in Box 1. There are some exemptions based on circumstances, for example, survivors of trafficking, or clinical condition, for example, some infectious diseases including HIV and COVID- 19. The charging regulations mandate that NHS trusts implement these charges and report any outstanding debts to the Home Office.The NHS overseas visitor charging policy forms one arm of a series of policies dubbed the Hostile Environment, which explicitly aims to make life untenable for those who are undocumented.^12^

A number of current initiatives seeking to address registration refusal focus on informing GP staff of current guidance and highlighting the reasons people may not have access to documentation.^15^^,^^16^ In order to increase uptake of COVID-19 vaccination, there have been increased efforts by local authorities, third-sector partners, and NHS England to encourage registration, including extensive reiteration of the guidance.^17^

Some have argued that it is the ‘drip down’ effect of secondary care policies restricting access to free care based on immigration status that leads to problems for people without documents registering with a GP.^18^^–^20 There is, however, a gap in understanding common practice around GP registration and why practice staff are not consistently following guidance. The authors found no research exploring the perspective of staff tasked with registering new patients. This makes it challenging to determine if existing initiatives are addressing all the drivers to exclusionary registration practice.

**Table:** How this fits in

Previous research shows that people are often refused GP registration if they do not have access to documentation, highlighting a discrepancy between guidance and practice that has not been previously explored. Current third-sector initiatives to improve inclusive registration have largely focused on reiterating guidance and explaining to staff why people may not have access to documentation. This study found that reluctance to register this group was common, and reluctance was generally fuelled not by lack of knowledge of the guidance, but by workplace and resourcing pressures, and moral judgements concerning who should be entitled to NHS services based on immigration status. The perceived practical and financial burdens relate to overall increases in workload and the current funding model utilised in general practice. Initiatives to improve access must acknowledge such concerns, alongside addressing the wider malignant impact of the Hostile Environment policies on individual staff decision making.

Access to care is a complex negotiation between individuals and healthcare services subject to multiple influences, at an individual and structural level. The concept of ‘candidacy’ can be used to understand the process that ‘vulnerable’ groups go through to access health care in the UK.^21^ A better understanding is needed of the ‘adjudication’ stage of access to GPs — that is, the judgements made by staff as to whether a person without documents can be registered. By exploring everyday registration practice, an understanding can be found of the processes through which registration might be refused, and the factors that operate to influence the application of the guidelines.

Two social theories are drawn on to analyse the phenomenon of GP registration without documentation, as they offer complementary lenses through which to consider everyday decision making by frontline workers. The first is Lipsky’s street-level bureaucracy,^22^ which considers the implementation of policy by ‘frontline’ staff, and the second is Bourdieu’s theory of practice, which offers a lens through which to situate the ‘practical logic’ of everyday life and decision making within structural power relations.^23^ Lipsky’s concept of public sector workers as ‘street-level bureaucrats’ (SLBs) seeks to explain the difference between written policy and how it is enacted on the ground. He argues that, in response to insurmountable demand and difficult working conditions, workers utilise ‘discretion’, and develop ways of interpreting and applying policy that reduce their work volume and complexity. He suggests that discretionary behaviour is informed by working conditions, rather than individual opposition to policy goals or deliberate subversion.^22^^,^^24^^,^^25^

A limitation of Lipsky’s theory is that drivers of ‘discretion’ are not socially situated.^26^^,^^27^ Therefore, Bourdieu’s theory of practice is utilised to help socially situate the experiences and perceptions of GP staff. Bourdieu’s ‘logic of everyday practice’ utilises the concepts of habitus, field, and capital to explain the relationship between an individual’s disposition and behaviour, and the structures and power dynamics of society.^23^

### Research aims

The aim of this research was to better understand the processes by which registration might be refused, and the factors that operate to influence this. This research explores participants’:

- accounts of their everyday practice;
- experience of obstacles to registration without documentation;
- perception of people without documentation; and
- key contextual factors that influence registration.

## METHOD

This qualitative study used a mixture of semi-structured interviews and focus groups (FGs) with patient assistants (PAs, also commonly referred to as receptionists), and practice managers (PMs) involved in new patient registration.

Participants were recruited from practices across three clinical commissioning groups (CCGs) in North East London. This decision was informed by estimates that London has the largest number of people with no fixed address and who are undocumented in the UK. Convenience sampling was applied as the research team had existing networks in North East London, allowing improved access to GP practices in these three CCGs. A total of 33 participants took part: 13 were interviewed individually and 20 took part in FGs (Table 1).

**Table 1.** Details of recruitment

**CCG**	**Participants (practices, *n*)**	**Method (participants, *n*)**	**Job role**
A	8 (3)	Individual interviews (8)	3 PMs, 5 PAs
B	14 (2)	2 FGs (6 and 8)	2 PMs, 12 PAs
C	11 (3)	Individual interviews (5) and 1 FG (6)	2 PMs, 9 PAs

*CCG = clinical commissioning group. FG = focus group. PA = patient assistant. PM = practice manager.*

The study was advertised by: 1) direct email from the research team to all practices in each CCG; 2) a second email cascaded by the CCG to all practices; 3) a study invite in CCG bulletins; and 4) a study invite distributed by community education provider networks.

Topic guides were developed with input from PAs during patient and public involvement, and adjusted iteratively. Data collection was completed by the first and second authors from April–November 2019. Braun and Clarke’s reflexive thematic analysis was applied, including their six- step approach.^28^ The data were audio-recorded, transcribed verbatim, and then coded by the first and second authors using NVivo software (version 12); themes were then generated.

Supplementary Appendix S1 has further detail of the methodology and about the research team’s positionality and reflexivity.

## RESULTS

Through this analysis the authors were able to identify everyday registration practices, and two distinct themes were generated related to staff reluctance to register those without documents: 1) those without documents as burdensome; and 2) deservedness and the queue.

Table 2 provides a key for the participant identity attached to quotes.

**Table 2.** Participant identity

**Participant information**	**Label next to quote**
CCG	3 × CCGs labelled A–C
Practice	7 × practices labelled 1–7
Participants	Labelled 1–33
Job role	PA = patient assistant, PM = practice manager
Type of data collection participant took part in	F = focus group, I = interview

*CCG = clinical commissioning group.*

### Everyday practice

Throughout participant discussions of everyday practice, it was clear that all but two participants were familiar with the relevant NHS guidance. However, the majority of participants expressed reluctance to register patients without documents. The minority of participants (two PMs and one FG) who did not express personal reluctance to register this group stated that they knew many practices local to them that refused to register this patient group.

The most common practice was to ask for formal proof of address and identification, and, if these could not be provided, to ask for increasingly less ‘formal’ documents including, for example, a letter from a family member:

*‘You must have a tenancy agreement, if you are living in somewhere. There has to be some trace of you … if the relative is registered here and they write a letter in on their behalf to say this person name, date of birth, is living with me, then we accept that … ’*

(A/1/2/PA/I)

In scenarios where no documents could be provided, only a small minority of participants were certain that they either would or would not register. The majority were unclear as to whether they would, despite attempts at clarification by interviewers. This may reflect variable practice, that it is an uncommon scenario, or that the participants did not want to share their working practice with interviewers.

Regarding perceptions of the guidance, only one participant, a PM, felt it was clear and easy to implement. The majority of participants felt it was ambiguous, leading to varied practice:

*‘I just think the guidelines are too ambiguous at the moment and that’s why people are doing it different ways.’*

(C/8/23/PM/I)

Participants drew attention to the contradiction in the guidance that you are allowed routinely to ask for documentation, and that there are instances where you may need to establish that someone is *‘who they say they are’*:^1^

*‘It’s a load of rubbish really isn’t it, at the end of the day, because you’re there telling that you ask for it, but in the other breath they are saying you can’t refuse.’*

(A/3/7/PA/I)

### Theme 1: Those without documents as burdensome

Participants from all practices characterised those without documents as burdensome; even the minority of participants who did not express reluctance registering this patient group felt that registering them placed a burden on their practice, which was probably why other practices were not following the guidance. Several participants, when talking about the burden of registering this patient group, also recognised that it is challenging for this group to access health care and important that they do so.

Patients without documents were characterised as ‘burdensome’, in multiple ways: administratively, financially, clinically, reputationally, and in relation to fears about safety and responsibility.

#### Administrative

Participants described that, when registering patients without documentation, the registration was more likely to be ‘rejected’ by Primary Care Support England’s (PCSE) central approval system as details were more likely to be incorrect or misspelt. They must then re-contact the patient to clarify their details, which increases workload. Some felt there was a contradiction between their duty of care to the individual and administrative responsibilities:

*‘We just want to care for the patient, as a human you know, we just want to look after them. We don’t want to do all this … but then again, to help our system and everything, proof of address and all the other information we ask, if we’ve got it helps us a lot. As a person as a human you want to help that person, but then you know you have to see both sides isn’t it.’*

(B/4/16/PA/FG)

#### Financial

Participants highlighted that those without documents were more likely to need translation services, and therefore double appointments, increasing costs to the practice. Additionally, it was felt they were likely to be difficult to contact, or move out of the area, making it more challenging to achieve Quality and Outcomes Framework (QOF) targets. One participant observed:

*‘A lot of practices focus on global sum payments and if they don’t get these from a quarter then it isn’t good enough for them … migrants do require a lot of attention and care with their health, catching up on immunisation, getting them up to speed … the GP does all they can and if you do it for two to three weeks costing you the equivalence of a whole year of another regular patient,* [then] *in two weeks’ time they are gone and you got paid nothing for it.’*

(C/10/30/PM/FG)

#### Clinical

Participants raised concerns about the increased amount of clinician time required by this patient group. One reason was due to increased appointment time needed:

*‘… because we register so many patients um that have difficulties registering elsewhere, so maybe because of their language difficulties or because they are still new to the country they are still learning the language, and so they don’t have the documentation to support where they live or whatever else, so we end up then having to give extra appointments because you have to give a double appointment for an interpreter, so that puts pressure on all of our other services and again, this is just down to us following the guidance.’*

(A/2/5/PM/I)

In addition, some participants perceived this group to be more likely to have complex needs (related to safeguarding or mental health) and that this would mean an increased frequency or length of clinical contact.

Within this theme, data relating to clinical burden were the least raised, which probably reflects that clinical staff were not included in this study.

#### Reputational

This was raised only by PM participants who felt that registering patients without documents affected their reputation, as the QOF system also acts as a measure of performance; the example given by several participants was that registering this patient group resulted in them performing more poorly for childhood vaccination rates. Some felt that following the guidance had resulted in registering disproportionate numbers of people without documents, compared with neighbouring practices, therefore furthering their disadvantage. One participant observed:

*‘I wouldn’t think that any practices are following that guidance … they get paid for hitting targets and anything that you do that might prevent you hitting targets is going to affect your income, so it’s just not going to happen.’*

(B/5/22/PM/FG)

#### Fears about safety and responsibility

Many participants expressed concern that patients without documents may be a risk to themselves, other patients, or staff. They felt that one reason patients may state they do not have documents is to prevent linkage to their correct medical record in order to conceal any risk they pose to others, or themselves. Even if identity was not purposefully concealed, being unable to link correct medical records, or receive correct information regarding address or identity, could mean that staff miss ‘red flags’ that they have a responsibility to pick up. They mentioned human trafficking, child safeguarding, and the Prevent duty. These concerns often seemed to be associated with possible scenarios rather than direct previous experience:

*‘I think for me the worst-case scenario would be where somebody has ended up suicidal and actually died because we didn’t have the full medical history because they didn’t give us the correct details.’*

(A/1/1/PM/I)

There was accompanying concern about who would be held responsible if harm occurred, and it was judged that document checking might have prevented it:

*‘It would be how did you not know this was a known paedophile and you allowed him to sit in a waiting room when you were doing a child imms clinic. You know or God forbid a terrorism act, or anything. I’m sure somewhere the blame would kind of come on us.’*

(A/1/1/PM/I)

While no anecdotal experience of such scenarios was reported by participants, the perception that checking documentation, particularly identity documents, was part of safeguarding procedure, providing protection from blame for the person registering, was expressed frequently. This is also woven into the guidance, which states that although documentation cannot be insisted on there are practical reasons to check people *‘are who they say they are’*.^1^ It does not elaborate as to what these reasons might be.

Regardless of practice around registration, and for multifaceted reasons, participants were mostly united in their perception of patients without documentation as a burden. The challenges in registering this patient group often arose from practical day-to-day administrative labour, but documentation was also perceived as part of safeguarding processes and preventing blame.

### Theme 2: Deservedness and the queue

Value judgements seemed to inform some participants’ reluctance to register patients without documents. Around one- third of participants raised concerns that people were fraudulently taking resources, or taking resources from those more ‘deserving’ of them, based on citizenship or contribution to ‘society’. This sentiment was not only expressed mostly in individual interviews, but also by two participants in two separate FGs; in one FG it went unchallenged and in the other there was general agreement. This may reflect participants expressing views in interviews that they would not feel comfortable expressing to colleagues.

They categorised possible reasons for not having documents into ‘genuine’, such as fleeing domestic violence or recent arrival in the country to join a spouse, and ‘not genuine’, such as being in the country unlawfully or concealing identity. This seemed in part due to difficulty understanding how someone could lack access to documentation:

*‘It is a fine line between the genuinely vulnerable people and people who are not but just flout the rules all the time.’*

(B/5/22/PM/FG)

*‘At the end of the day, we’re just trying to make sure they’re not coming, you know, illegally or something.’*

(B/4/9/PA/FG)

*‘I just think you must have something, you know when you think, you must have a bit of paper with your name and address on it somewhere … um* [pause] *that’s the only thing. You can’t live here, in this world, and not be picked up by something.’*

(A/1/3/PA/I)

Registration was seen as the first step in accessing the NHS, including those services a patient may not be entitled to in secondary care. Some participants felt that those without documents, whose NHS entitlement was restricted because of their immigration status, were less deserving of healthcare resources than those with citizenship. They expressed the view that, in a system with increasingly finite resources, contribution to the system should inform your place in the queue:

*‘There’s people coming from all over the world, who can come here, get treatment, lie through their teeth, owe the NHS thousands of pounds, and you’ve got genuine people who have worked all their lives and can’t get any treatment, got to wait months or years for it. It does hurt I am afraid.’*

(A/3/8/PA/I)

## DISCUSSION

### Summary

It was found that lack of knowledge of guidance did not explain reluctance to register people without documents. The findings show that reluctance is driven by:

1. Pragmatic concerns regarding burden on services from multiple perspectives: administrative, financial, reputational, safeguarding, and with regard to concern about personal responsibility.
2. Moral judgements that being unable to produce documents may signal: identity fraud, hiding a violent past, or not being a ‘legal’ citizen; conditions that conferred a sense of ‘undeservingness’ of scarce NHS resources.
3. Wider structural factors and policies: moral concerns were raised in the context of access restrictions to secondary care. This perhaps reflects a ‘trickle down’ impact of the overseas visitors charging policy and broader popular anti-migration sentiments articulated by politicians and the media.

### Strengths and limitations

The use of qualitative methodology facilitated the collection of rich data, which was key to revealing complex phenomena and explanatory models. These data provide new insights to inform initiatives to improve inclusive registration practice, particularly locally in North East London. Efforts were made to ensure that data collection and analysis were robust through participant involvement and collective team reflection.

Transferability is limited by a number of factors:

1. Participants focused mostly on people who were undocumented, in particular migrants, with little discussion of other minority groups, such as the homeless and GRT communities (interviewers did not define who those without documents might be, nor provide prompts to participants related to groups not discussed).
2. Sampling was not purposive or representative. Invitations were cascaded via PM or generic practice emails and may not have reached all staff.
3. As the aim of the study was to improve inclusive registration practice, this may have influenced who volunteered to participate and what they chose to share in data collection. However, participants did appear to speak openly about both their reluctance to register patients without documents, and their ideological disagreements with current practice related to migration status and healthcare access.

As detailed in the method section, FGs included both PAs and PMs, which introduced possible significant power dynamics into these groups, which may have restricted what PAs felt able to discuss.

Data collection occurred before the COVID-19 pandemic; therefore, changes to registration practice following this are not considered in this research. In qualitative research conducted during the pandemic considering access to primary care for people who have migrated, healthcare workers described a shift towards digitisation of registration, and some study participants felt that this may have reduced access to care more generally, as well as making it harder to register with a GP for this specific group.^29^ Data were not available on the proportion or geographical location of practices that allow digital registration, nor how this changed during COVID-19. However, and possibly counter to the above, some digital registration mechanisms do not ask for any proof of address or identification, and therefore may actually represent an easier route to registration for some; this is an important area of further research.

### Comparison with existing literature

To the authors’ knowledge, this is the first study to consider the registration of those without documents from the viewpoint of those staff tasked with registering them. Previous initiatives to improve inclusive registration^15^^,^^16^ have focused on staff misunderstandings of either the guidance or patient group; however, this research has revealed alternative understandings requiring complex intervention.

Lipsky’s SLB theory^22^ argues that, in response to difficult working conditions, workers utilise discretionary behaviours to reduce the volume and complexity of their work. This was evident in participants’ reluctance to enact registration policy, stemming from a perception of this patient group as burdensome, a need to reduce workload, and to protect themselves from blame. However, this research reveals that reluctance to register was not only driven by participants’ working conditions as Lipsky theorises, but also by moral judgements about who should be prioritised for limited healthcare resources.

These findings echo other critiques of Lipsky that argue discretionary behaviour is not just an act of self-preservation but is also influenced by values, norms, culture, and a sense of belonging to social groups.^26^^,^^27^ For example, Maynard-Moody and Musheno, in their study of public sector workers, observed that discretionary behaviour was informed primarily by individual value judgements about clients rather than concerns regarding workload.^26^ Furthermore, the critical interpretive synthesis of the literature on healthcare access for vulnerable groups, which the candidacy framework was born from, suggests healthcare workers’ perceptions of ‘social deservingness’ informs decisions to allow access, in keeping with the findings here.^21^

Bourdieu’s ‘logic of everyday practice’ offers an analysis of these individual value judgements that extends beyond the consideration of limited resources to the wider structures and power dynamics of society, building on Lipsky’s original characterisation of the SLB.^22^ Bourdieu illuminates the relationship between individual front-desk behaviour including moral judgements, and the broader social spaces that we occupy; in this instance, these are the NHS and its policies restricting access to public services. It was found that participants’ individual moral judgements clearly mirrored broader health policy restrictions to secondary care based on immigration status, and popular political ideology around citizenship and access to public services. Kang *et al* have similarly found that Hostile Environment policies in secondary care have compounded barriers to accessing primary care.^9^

Bourdieu’s ‘habitus’ refers to the socialised norms guiding people’s behaviour, which often feels like a ‘gut instinct’ rather than something influenced by structural power dynamics or individual opposition to policy goals.^23^ This seemed to be reflected in some research participants’ strong ‘sense’ of who deserves access to finite NHS resources, reproducing the values of ‘undeservedness’ and the Hostile Environment.

The perceived ambiguity of registration guidance and the confusion created by contradictory eligibility requirement between primary and secondary care perhaps leaves decisions particularly vulnerable to personal interpretation.

Participant descriptions of managing the tension between top-down policies intended to improve inclusive registration, and those restricting secondary care based on immigration status, reflected Bourdieu’s conceptualisation of the *‘left and right hand of the state’* .^30^ The ‘left hand’ refers to the provision of welfare while the ‘right hand’ refers to restrictive processes of the state. Berlin *et al* identified the *‘painful personal tension’* for healthcare workers providing care to the individual while sustaining a health system with limited resources.^31^ In this research, this tension was evident in a common concern that, in prioritising their duty of care to the person in front of them, participants may disadvantage more ‘deserving’ patients.

In conclusion, many participants described a reliance on ‘instinctual’ individual decision making at the GP front desk based on local resources, normalised practices, and constructions of social ‘deservedness’, which may result in disadvantaging people who cannot access documentation.

### Implications for research and practice

Participants in this study shared narratives revealing the barriers to registering potentially vulnerable groups. These barriers extend beyond a lack of knowledge of guidance. Understanding these complex and sometimes competing narratives is necessary to improve efforts to increase adherence to inclusive registration practice by appreciating the reality of the difficult work front-desk staff are tasked with. SLB theory reminds us that attempts to control discretionary behaviour, in particular through reiterations of top-down guidance, will likely be futile.^21^^,^^22^ While future initiatives need to address staff concerns around culpability, workload, and funding, a Bourdieusian lens reveals the impact of societal power dynamics on everyday workplaces, serving as a reminder that we must also tackle the wider impact of the Hostile Environment on individual staff decision making in general practice.
